# Association of post-operative CEA with survival and oxaliplatin benefit in patients with stage II colon cancer: a post hoc analysis of the MOSAIC trial

**DOI:** 10.1038/s41416-019-0521-7

**Published:** 2019-07-12

**Authors:** Edouard Auclin, Thierry André, Julien Taieb, Maria Banzi, Jean-Luc Van Laethem, Josep Tabernero, Tamas Hickish, Aimery de Gramont, Dewi Vernerey

**Affiliations:** 1 Department of Hepato-Gastroenterology and Gastrointestinal Oncology, Sorbonne Paris-Cité, Paris Descartes University, Hôpital Européen Georges Pompidou, Paris, France; 2 Methodology and Quality of Life Unit in Oncology, University Hospital of Besançon, Besançon, France; 3 Bourgogne Franche-Comté University, INSERM, EFS BFC, UMR1098, Interactions Hôte-Greffon-Tumeur/Ingénierie Cellulaire et Génique, Besançon, France; 4 Department of Medical Oncology, Sorbonne University, Hôpital Saint Antoine, Paris, France; 5 Oncology Multidisciplinary Research Group (GERCOR), Paris, France; 6 UMR-S 1147, INSERM, Paris, France; 7 Unit of Medical Oncology, Clinical Cancer Center, AUSL-IRCCS Reggio Emilia, Reggio Emilia, Italy; 8 Department of Gastroenterology and Digestive diseases, Hopital Erasme, Université Libre de Bruxelles, Bruxelles, Belgium; 9 Vall d’Hebron University Hospital and Institute of Oncology (VHIO), CIBERONC, Universitat Autònoma de Barcelona, Barcelona, Spain; 10 Department of Oncology, Royal Bournemouth Hospital and Bournemouth University, Bournemouth, UK; 11 Department of Oncology, Institut Hospitalier Franco-Britannique, Levallois-Perret, France

**Keywords:** Colorectal cancer, Colon cancer

## Abstract

**Background:**

Adjuvant treatment for stage II colon cancer (CC) can be proposed to patients with high-risk disease. Recently, 2.35 ng/mL carcinoembryonic antigen (CEA) was identified as the best cut-off value. This post hoc analysis of the MOSAIC trial assessed post-operative CEA prognostic value for survival outcomes and predictive value for the addition of oxaliplatin to adjuvant treatment.

**Methods:**

Prognostic and predictive values of post-operative CEA in patients with stage II CC were evaluated with Kaplan–Meier survival curves and Cox model with interaction terms. Disease-free survival (DFS) and overall survival (OS) were estimated.

**Results:**

Among 899 stage II CC patients, post-operative CEA was available in 867 (96.4%); and 434 (48.65%) had a high-risk stage II disease. The 3-year DFS rate was 88.5% and 78.7% in the ≤ 2.35 ng/mL and > 2.35 ng/mL group, respectively (*P* = 0.006). Use of oxaliplatin showed survival benefit only in patients with high-risk stage II CC and post-operative CEA > 2.35 ng/ml (interaction term *P* = 0.09 and 0.03 for DFS and OS).

**Conclusion:**

CEA is a strong prognostic factor for DFS and OS in stage II CC. In the MOSAIC trial, only high-risk stage II CC patients with post-operative CEA > 2.35 ng/mL benefited from the addition of oxaliplatin to LV5FU2.

**Trial registration:**

NCT00275210 (January 11, 2006).

## Background

Colon cancer (CC) is the third most common cancer in men and women.^1,2^ Seventy-five percent of the patients are diagnosed at a localised stage; where a curative treatment can be proposed. Although adjuvant chemotherapy after curative surgery is recommended for stage III CC patients,^3^ its survival benefit for stage II CC is still debated. In this latter group of patients, adjuvant fluoropyrimidine-based chemotherapy with or without oxaliplatin is used. The results of subgroups analyses of large adjuvant trials and meta-analysis have demonstrated several risk factors for recurrence or death in stage II CC that guide adjuvant treatment decisions.^3^ In the MOSAIC trial, the estimated 10-year probability of overall survival (OS) was 75.4% for FOLFOX4 (5-fluorouracil, leucovorin, and oxaliplatin) and 71.7% for LV5FU2 (fluorouracil and leucovorin)^4,5^ in high-risk CC patients with a simplified definition of high-risk stage II disease (T4, tumour perforation, or <10 examined lymph nodes). In this study, the addition of oxaliplatin to LV5FU2 did not provide any survival benefit for low-risk stage II CC patients. Nowadays, there is a need to better identify patients for whom the addition of oxaliplatin to LV5FU2 can reduce disease relapse or death, but also to limit useless long-lasting toxicities induced by oxaliplatin.

Carcinoembryonic antigen (CEA) is a well-known low cost biological tumour marker used in CC since 1965.^6^ Previous large studies have shown that elevated pre-operative CEA levels are associated with worse prognosis in patients with stage I and II disease, who did not receive adjuvant chemotherapy, and for whom pre-operative CEA value of 2.35 ng/mL was defined as an optimal cut-off point for survival stratification.^7^

The aims of this post hoc study were (i) to assess and validate precisely the prognostic value of post-operative CEA for disease-free survival (DFS) and OS in patients with stage II CC treated by adjuvant chemotherapy and (ii) to determine the additional predictive value of post-operative CEA for the benefit of the addition of oxaliplatin to LV5FU2.

## Methods

### Population

All stage II CC patients from the MOSAIC phase III trial (NCT00275210) were included in this post hoc analysis.^4^ Post-operative CEA <10 ng/mL was an inclusion criterion in the MOSAIC trial. The CEA measurement was not centralised.

### Definition of high-risk stage II CC

High-risk stage II CC was defined as being characterised by one of the following factors: T4, tumour perforation, and <10 or <12 examined lymph nodes (the MOSAIC definition and the modified MOSAIC definition, respectively).

### Statistical analysis

Median values (interquartile range) and frequencies (percentage) were provided for the description of continuous and categorical variables, respectively. Medians and proportions were compared using Student’s *t*-test and chi-square test (or Fisher’s exact test, if appropriate), respectively.

DFS was defined as the time between randomisation and local/distant relapse, second colorectal/rectal occurrence, or death, whichever occurred first. Alive patients without relapse and second colorectal/rectal cancer were censored at the date of their last follow-up.

OS was defined as the time between randomisation and death from any cause. Patients known to be alive were censored at the date of their last follow-up.

DFS and OS were estimated using the Kaplan–Meier method and described using median or rate at specific time points with their 95% confidence intervals (95% CIs). Follow-up was calculated using the reverse Kaplan–Meier method.

When used continuously, the association between CEA and survival was investigated with the restricted cubic splines method with graphical evaluation. When used as a categorical variable, the CEA cut-point defined by Margalit et al. in a cohort of 45 449 stage I-II CC patients was applied. A sensitivity analysis was performed with the Horton and Lausen method to find the best CEA cut-off for risk stratification in our cohort.^8^

The association of demographic, clinical, biological and molecular factors with survival was first assessed by the univariate Cox-proportional-hazard model, providing hazard ratios (HRs) and 95% CIs. Parameters with *P*-values of <0.10 in univariate analysis and/or clinically relevant variables were entered into the multivariable Cox-regression model.

A differential DFS and OS treatment effect among the identified CEA risk groups but also when considering the CEA risk groups identified as an additional parameter to include in classical well-established risk group definitions was evaluated with an interaction term in the Cox-regression model and illustrated with Kaplan–Meier curves.

All analyses were performed using SAS version 9.4 (SAS Institute, Cary NC) and R software version 2.15.2 (R Development Core Team, Vienna, Austria; http://www.r-project.org). *P*-values of <0.05 were considered statistically significant and a threshold of 0.1 was used for interaction terms. All tests were two-sided.

## Results

### Characteristics of patients

Among the 2246 patients included in the MOSAIC trial, 899 had stage II CC. Post-operative CEA was available in 867 (96.4%) patients; 834 (96.2%) had CEA <5 ng/mL and 664 (76.6%)  <2.35 ng/mL.

Overall, 434 (48.65%) and 520 (58.3%) patients had high-risk stage II CC according to the MOSAIC and the modified MOSAIC definition, respectively (Table 1). The MSI status was well balanced between patients with CEA <2.35 and >2.35 ng/mL (12.4% vs 16.3%, *P* = 0.36).

**Table 1:** Patient characteristics

		MOSAIC Trial Stage II CC patients (*N* = 899)
Age at inclusion, years	≤ 70	798 (88.77%)
	> 70	101 (11.23%)
Gender^a^	Female	407 (45.27%)
	Male	492 (54.73%)
Body mass index^a^	Underweight	27 (3%)
	Normal	438 (48.72%)
	Overweight	324 (36.04%)
	Obese	110 (12.24%)
Type of adjuvant chemotherapy^a^	FOLFOX4	451 (50.17%)
	LV5FU2	448 (49.83%)
Tumour location^a^	Left	580 (64.52%)
	Right	319 (35.48%)
Histoprognostic grade	G1/2	763 (89.55%)
	G3/4	89 (10.45%)
	Missing	47
Stage II risk group	High^b^	434 (48.65%)
(MOSAIC definition)	Low^c^	458 (51.35%)
	Missing	7
Stage II risk group	High	520 (58.3%)
(modified MOSAIC definition)	Low	372 (41.7%)
	Missing	7
T-stage^a^	T3	728 (80.98%)
	T4	171 (19.02%)
Number of nodes examined^a^	Median (IQR)	12 (8–19)
Performance status*	0–1	799 (88.9%)
	≥ 2	100 (11.1%)
Bowel perforation^a^		81 (9.01%)
Bowel obstruction^a^		158 (17.58%)
Vascular invasion	Yes	80 (16.29%)
	Missing	408
MMR status	dMMR	48 (13.15%)
	pMMR	317 (86.85%)
	Missing	534
CEA level, ng/mL	Median (IQR)	1.4 (0.9–2.2)
standard CEA cut-off level, ng/mL	≤ 5	834 (96.19%)
	> 5	33 (3.81%)
CEA cut-off level reported by Margalit et al., ng/mL	≤ 2.35 > 2.35	664 (76.6%) 203 (23.4%)
CEA cut-off level according to Hothorn method, ng/mL	≤ 2.77 > 2.77 Missing	724 (83.51%) 143 (16.49%) 32
Time between surgery and CEA measurement, weeks	Median (IQR)	4.4 (3.3–5.4)
Follow-up, years Median	Median	8.8 (7.9–9.5)

*CC* colon cancer, *MMR* mismatch repair, *CEA* carcinoembryonic antigen, *CI* confidence interval, *IQR* interquartile range

^a^no missing data

^b^T4, tumour perforation, or fewer than 10 lymph nodes examined

^c^T1–3 and no tumour perforation and 10 or more lymph nodes examine

### Post-operative CEA and survival

**Fig. 1:**
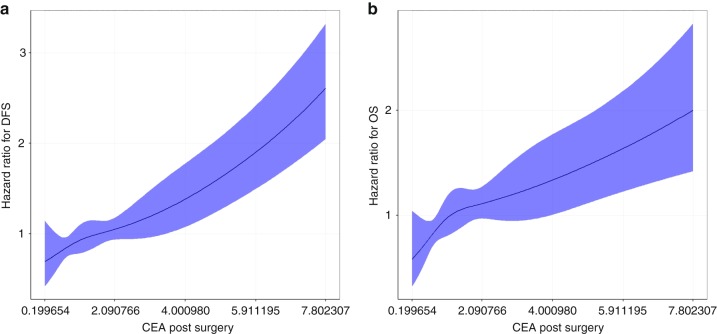
Association between CEA and DFS (**a**) or OS (**b**) by the restricted cubic splines method

When post-operative CEA was used as continuous variable, a gradual risk suggesting a linear relation between post-operative CEA and DFS and OS was identified (Fig. 1). Different risk populations among patients with a post-operative CEA level of <5 ng/mL were determined. This result validates in our cohort the lower cut-off of 2.35 ng/mL found by Margalit et al.

**Fig. 2:**
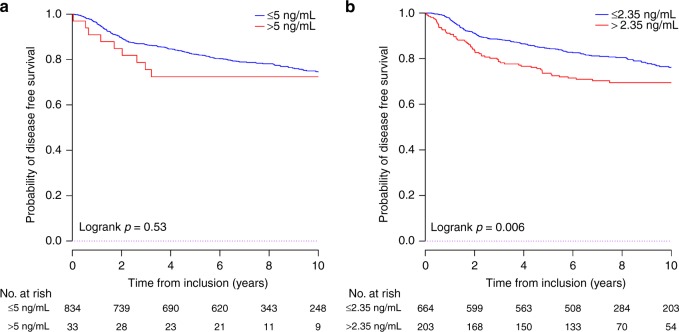
DFS according to (**a**) CEA 5 ng/mL and CEA 2.35 ng/mL (**b**)

When post-operative CEA was used as a categorical variable, a strong trend was observed between ≤5 and >5 ng/mL values and DFS, with the 3-year DFS rate of 86.6% and 75.5% in the ≤ 5 and > 5 ng/mL groups, respectively; *P* = 0.53. However, the difference was not significant (Fig. 2a).

When using the 2.35 ng/mL cut-off identified by Margalit et al, patients with a post-operative CEA level ≤2.35 ng/mL were at lower risk of recurrence or death. The 3-year DFS rate was 88.5 and 78.7% for patients with CEA ≤2.35 ng/mL and >2.35 ng/mL, respectively (*P* = 0.006; Fig. 2b).

Similar results were observed with OS (Supplementary Fig. S1). In the sensitivity analysis, a threshold value of 2.77 ng/mL was identified as the optimal CEA cut-off (Supplementary Figs. S2 and S3).

### Independent prognostic value of post-operative CEA

In univariate analysis, a post-operative CEA level of >2.35 ng/mL was associated with DFS and OS (DFS: HR 1.51, 95% CI 1.12–2.03; *P* = 0.006 and OS: HR 1.46, 95% CI 1.05–2.05; *P* = 0.03).

After adjustment for age, gender, tumour location, bowel obstruction, and MOSAIC risk group, CEA >2.35 ng/mL was still significantly associated with DFS (HR 1.49, 95% CI 1.10–2.00; *P* = 0.009) and OS (HR 1.90, 95% CI 1.33–2.72; *P* < 0.0001; Table 2 and Supplementary Table S1).

**Table 2:** Multivariate Cox-regression analysis of DFS (*N* = 860)

		HR	95% CI	*P*
Age, years	>70	1.94	1.37–2.76	<0.0001
Gender	Male	1.35	1.02–1.79	0.035
Tumour location	Right	0.78	0.58–1.06	0.111
MOSAIC risk group	Low	0.74	0.56–0.98	0.035
Bowel obstruction	Yes	1.48	1.07–2.03	0.016
CEA level, ng/mL	> 2.35	1.49	1.10–2.00	0.009

*HR* hazard ratio, *CI* confidence interval, *CEA* carcinoembryonic antigen

### Predictive value of post-operative CEA

**Fig. 3:**
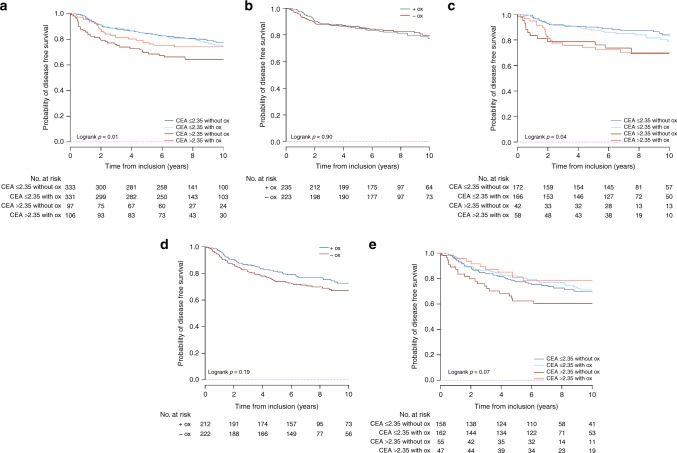
Benefit for DFS with the addition of oxaliplatin to LV5FU2 in **a** all patients, **b** the MOSAIC low-risk patients, **c** the MOSAIC low-risk patients after CEA stratification, **d** the MOSAIC high-risk patients, and **e** the MOSAIC high-risk patients after CEA stratification. Figure 3 abbreviations: Ox oxaliplatin; +ox with oxaliplatin; Ox without oxaliplatin

The benefit of the addition of oxaliplatin to LV5FU2 in term of DFS and OS was different between the two CEA groups (Table 3 and Supplementary Table S2). Only patients with high post-operative CEA levels seemed to have survival benefit from oxaliplatin addition to LV5FU2 (interaction terms *P* = 0.09; Fig. 3a).

**Table 3:** Disease-free survival according to treatment arm and CEA level

DFS									
				3 y % (95% CI)					*P-*value for the interaction term between CEA (≤ 2.35, > 2.35) and Treatment Arm (LV5FU2, FOLFOX)
	All *N*	LV5FU2 arm *N*	FOLFOX arm *N*	LV5FU2 arm	FOLFOX arm	Absolute change^d^	Relative change^e^	HR for treatment effect^c^ (95% CI)	
Whole population	899	448	451	84.7 (81.4–88.1)	87.1 (84–90)	+2.4	+2.8		0.11
CEA ≤2.35	664	333	331	88.2 (84.8–91.7)	88.7 (85.4–92.2)	+0.5	+0.6	1.07 (0.77–1.47)	
CEA >2.35	203	97	106	76 (67.9–85.1)	81.1 (74–88.9)	+5.1	+6.7	0.67 (0.41–1.11)	
High-risk^a^ population	434	222	212	81.3 (76.2–86.6)	86.3 (81.7–91)	+5	+6.1		0.09
High-risk - CEA ≤2.35	320	158	162	84 (78.4–89.9)	86.3 (81.2–91.8)	+2.3	+2.7	0.95 (0.62–1.45)	
High-risk - CEA >2.35	102	55	47	74 (63.2–86.7)	87.2 (78.2–97.3	+13.2	+17.8	0.47 (0.23–0.97)	
Low-risk^b^ population	458	223	235	87.9 (83.7–92.3)	88 (83.9–92.3)	+0.1	+0.1		0.78
Low-risk - CEA ≤2.35	338	172	166	91.8 (87.8–96)	91.5 (87.3–95.8)	−0.3	−0.3	1.18 (0.71–1.95)	
Low-risk - CEA >2.35	100	42	58	78.6 (67.1–92)	75.8 (65.6–87.7)	−2.8	−3.6	0.99 (0.47–2.10)	

Absolute difference at time X gives the difference between percentages observed in the two treatment arms at time X; the relative difference at time X gives the proportion of increase or decrease in survival rate of one arm relative to the other arm at time X

^a^T4, tumour perforation, or fewer than 10 lymph nodes examined

^b^T1–3 and no tumour perforation and 10 or more lymph nodes examined

^c^HR for treatment effect (the addition of oxaliplatin to the LV5FU2 regimen)

^d^Absolute difference reflects a comparison of survival between the FOLFOX and LV5FU2 arms

^e^Relative difference reflects a ratio of the observed survival in the FOLFOX arm and the LV5FU2 arm [(X year OS rate in the FOLFOX group - X year OS rate in

the LV5FU2 group)/(X year OS rate in the LV5FU2 group) *100]

Among patients with high post-operative CEA levels, the benefit from oxaliplatin addition was observed in the high-risk stage II CC patients (DFS interaction term *P* = 0.09 and OS interaction term *P* = 0.03), but not in those with low-risk stage tumours (DFS interaction term *P* = 0.78 and OS interaction term *P* = 1). Patients with high-risk stage II tumours and CEA >2.35 ng/mL represent 25% of all stage II patients included in this post hoc analysis. The addition of oxaliplatin had no survival advantage in patients with low-risk stage II disease and CEA < 2.35 ng/mL (75% of the study population; Fig. 3c–e; Table 3 and Supplementary Table S2).

**Fig. 4:**
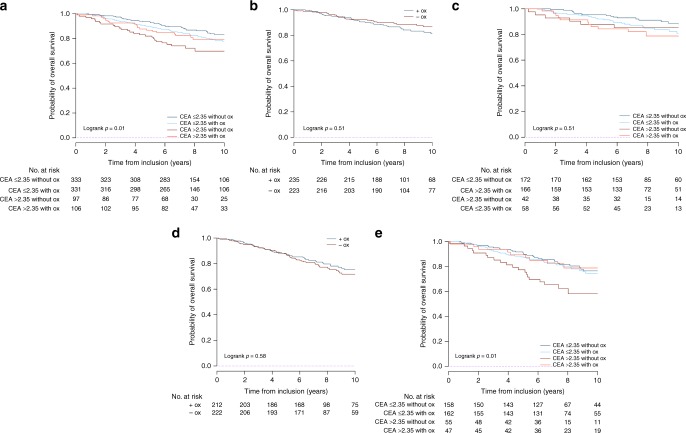
Benefit for OS with the addition of oxaliplatin to LV5FU2 in (**a**) all patients, (**b**) the MOSAIC low-risk patients, (**c**) the MOSAIC low-risk patients after CEA stratification, (**d**) the MOSAIC high-risk patients, and (**e**) the MOSAIC high-risk patients after CEA stratification. Figure 4 *abbreviations: Ox: oxaliplatin;* *+* *ox: with oxaliplatin; Ox: without oxaliplatin*

These results were replicated for the OS endpoint (Fig. 4) and using the modified MOSAIC definition for risk groups (Supplementary Figs. S4 and S5). When assessed in the population aged ≤70 years, the predictive value of CEA level was also observed in the high-risk group (Supplementary Figs. S6 and S7).

Similar results were observed with the optimal CEA cut-off value of 2.77 ng/mL (Supplementary Table S3).

## Discussion

In this post hoc analysis of the MOSAIC study, post-operative serum CEA level, with the cut-off of 2.35 ng/mL, was an independent prognostic factor in CC patients with stage II disease. Moreover, CEA appeared to be a predictive factor of the benefit from the addition of oxaliplatin to LV5FU2 in adjuvant therapy for high-risk stage II CC.

The prognostic value of post-operative CEA has been demonstrated for stage I to IV CC.^9^ Our results are in line with the literature, as we also showed that a lower cut-off of CEA is more suitable level for death or recurrence risk stratification. The cut-off values of 2.35 ng/mL (Margalit et al. study)^7^ and 2.77 ng/mL (defined in our cohort study) suggest that the common 5 ng/mL cut-off is clearly not optimal for risk stratification of patients with CC. The magnitude of the prognostic value of the CEA level was important in our study. Indeed, patients with high level of CEA had a 50% increased risk of death or recurrence compared to those who had CEA <2.35 ng/mL in multivariate analysis. We analysed the post-operative CEA level given the results by Konishi et al. who showed that the pre-operative CEA level is not a relevant marker of recurrence in localised CC if CEA is normalised after surgery.^10^

We showed in our study that only patients with high-risk stage II CC and high post-operative CEA level benefited from the addition of oxaliplatin in terms of DFS, with a 13% absolute increase in 3-year DFS rate. Another predictive factor of oxaliplatin benefit that has been published, is a high-risk score with the Oncotype Dx genomic signature.^11^ In fact, Yothers et al. showed, in a limited cohort of patients, that only stage II CC with high risk Oncotype Dx genomic signature seemed to benefit from the addition of oxaliplatin to adjuvant therapy.

Circulating tumour DNA (ctDNA) has shown remarkable prognostic value for OS and RFS in very small cohorts of patients with early-stage CC.^12–14^ Lu et al. suggested that the combination of post-operative serum CEA levels assessment and persistent post-operative circulating tumour cells detection is prognostic predictor of early relapse in stage II–III CC patients.^15^

All the above results suggest that a minimal residual disease may be associated with ctDNA, circulating tumour cells, CEA, or prognostic genomic signatures and that patients harbouring these factors are those who can benefit most from treatment intensification.

In our study, we used the risk groups definition published by André et al in 2015 (T4, tumour perforation, or less than 10 examined lymph nodes). This definition was published in the MOSAIC population, in order to lose as less information as possible. As it is slightly different from the ESMO consensus definition (12 lymph nodes examined instead of 10), we also validated our results with the latter definition (modified MOSAIC definition). Moreover, in order to deal with potential confounding factors related to comorbidities, we performed sensitivity analysis in the ≤70 years’ population. Again, similar results were observed, adding to their reliability.

In addition, in patients with high-risk stage II CC and post-operative CEA >2.35 ng/mL most deaths occurred within 2 years after surgery. This strongly suggest that post-operative imaging including PET-scan should be performed in those patients in order to further look at metastases before engaging adjuvant therapy, especially in randomised clinical trials.

Our results are robust and obtained with the best material available to study the interest to consider post-operative CEA information among classical high and low-risk classification in order to better identify patients who can benefit most from the addition of oxaliplatin to LV5FU2. Ideally, our findings should be validated in an external cohort data. Unfortunately, such data do not exist. Indeed, no randomised trial that have studied the addition of oxaliplatin to LV5FU2 in stage II CC patients provide precise post-operative CEA data. Another limit of this study is the lack of smoking habit data, a possible confusion factor. Therefore, clinician should interpret these results with caution in smoking patients.

## Conclusion

Our results show that post-operative CEA is a strong prognostic factor for DFS and OS in stage II CC. In the MOSAIC trial, only high-risk stage II CC with post-operative CEA >2.35 ng/mL (~25% of our stage II population) were identified to benefit from the addition of oxaliplatin to LV5FU2. CEA >2.35 ng/mL should be included in the definition of high-risk stage II CC and should lead to post-operative check-up before adjuvant therapy. Moreover, it should be included in the future trials assessing adjuvant strategies in stage II CC.

## Supplementary information

Supplemental Material

## Data Availability

Dataset of the study can be found with the corresponding author.

## References

[1] Siegel RL, Miller KD, Jemal A (2018). Cancer statistics, 2018. CA. Cancer J. Clin..

[2] Malvezzi M, Bertuccio P, Levi F, La Vecchia C, Negri E (2014). European cancer mortality predictions for the year 2014. Ann. Oncol..

[3] Labianca R, Nordlinger B, Beretta GD, Mosconi S, Mandalà M, Cervantes A (2013). Early colon cancer: ESMO clinical practice guidelines for diagnosis, treatment and follow-up. Ann. Oncol..

[4] André T, Boni C, Mounedji-Boudiaf L, Navarro M, Tabernero J, Hickish T (2004). Oxaliplatin, fluorouracil, and leucovorin as adjuvant treatment for colon cancer. N. Engl. J. Med..

[5] André T, de Gramont A, Vernerey D, Chibaudel B, Bonnetain F, Tijeras-Raballand A (2015). Adjuvant fluorouracil, leucovorin, and oxaliplatin in stage II to III Colon cancer: updated 10-year survival and outcomes according to BRAF mutation and mismatch repair status of the MOSAIC study. J. Clin. Oncol..

[6] Gold P, Freedman SO (1965). Specific carcinoembryonic antigens of the human digestive system. J. Exp. Med..

[7] Margalit O, Mamtani R, Yang Y-X, Reiss KA, Golan T, Halpern N (2018). Assessing the prognostic value of carcinoembryonic antigen levels in stage I and II colon cancer. Eur. J. Cancer.

[8] Hothorn, T. & Lausen, B. On the exact distribution of maximally selected rank statistics. *Comput. Stat. Data. Anal.* **43**, 121–137 (2003).

[9] Thirunavukarasu P, Sukumar S, Sathaiah M, Mahan M, Pragatheeshwar KD, Pingpank JF (2011). C-stage in colon cancer: implications of carcinoembryonic antigen biomarker in staging, prognosis, and management. J. Natl. Cancer. Inst..

[10] Konishi T, Shimada Y, Hsu M, Tufts L, Jimenez-Rodriguez R, Cercek A (2018). Association of preoperative and postoperative serum carcinoembryonic antigen and colon cancer outcome. JAMA Oncol..

[11] Yothers G, O’Connell MJ, Lee M, Lopatin M, Clark-Langone KM, Millward C (2013). et al. Validation of the 12-gene colon cancer recurrence score in NSABP C-07 as a predictor of recurrence in patients with stage II and III colon cancer treated with fluorouracil and leucovorin (FU/LV) and FU/LV plus oxaliplatin. J. Clin. Oncol..

[12] Fan G, Zhang K, Yang X, Ding J, Wang Z, Li J (2017). Prognostic value of circulating tumor DNA in patients with colon cancer: systematic review. PLoS ONE..

[13] Lecomte T, Berger A, Zinzindohoué F, Micard S, Landi B, Blons H (2002). Detection of free-circulating tumor-associated DNA in plasma of colorectal cancer patients and its association with prognosis. Int. J. Cancer.

[14] Tie J, Wang Y, Tomasetti C, Li L, Springer S, Kinde I (2016). Circulating tumor DNA analysis detects minimal residual disease and predicts recurrence in patients with stage II colon cancer. Sci. Transl. Med..

[15] Lu C-Y, Uen Y-H, Tsai H-L, Chuang S-C, Hou M-F, Wu D-C (2011). Molecular detection of persistent postoperative circulating tumour cells in stages II and III colon cancer patients via multiple blood sampling: prognostic significance of detection for early relapse. Br. J. Cancer.

