# Supplementary material for: Association of post-operative CEA with survival and oxaliplatin benefit in patients with stage II colon cancer: a post hoc analysis of the MOSAIC trial
Source: Br J Cancer. 2019 Jul 12;121(4):312–7. doi: 10.1038/s41416-019-0521-7 (PMC6738041; doi:10.1038/s41416-019-0521-7)
Supplement: Supplementary file 1 — Supplemental Material [file 41416_2019_521_MOESM1_ESM.docx]

**SUPPLEMENTARY TABLES**

**Sup Table S1:** Multivariate Cox regression model for OS (*N*=860)

|  |  | HR | 95% CI | *P* |
| --- | --- | --- | --- | --- |
| Age, years | > 70 | 2.06 | 1.40-3.04 | <0.0001 |
| Gender | Male | 1.50 | 1.09-2.07 | 0.013 |
| Tumor location | Right | 0.76 | 0.54-1.07 | 0.117 |
| MOSAIC risk group | Low | 0.66 | 0.48-0.91 | 0.011 |
| Bowel obstruction | Yes | 1.64 | 1.16-2.33 | 0.005 |
| CEA level, ng/mL | > 2.35 | 1.45 | 1.04-2.04 | 0.029 |

*Abbreviations: HR, hazard ratio; CI, confidence interval; CEA, carcinoembryonic antigen*

**Sup Table S2**: The 3-year OS according to treatment arm and CEA level

|  |  | |  |  |  | ***P* value for the interaction term between CEA (≤ 2.35, >2.35) and Treatment Arm (LV5FU2, FOLFOX)** |
| --- | --- | --- | --- | --- | --- | --- |
|  | **LV5FU2**  **arm** | **FOLFOX**  **arm** | **Absolute change** | **Relative change** | **HR for treatment effect* (95%CI)** |  |
| **Whole population** | 94.6 (92.5-96.7) | 93.7 (91.5-96) | -0.9 | -0.9 |  | 0.03 |
| - CEA ≤ 2.35 | 97.3 (95.5-99) | 93.9 (91.3-96.5) | -3.4 | -3.5 | 1.28 (0.89-1.85) |  |
| - CEA > 2.35 | 88.4 (82.2-95.1) | 92.5 (87.6-97.6) | +4.1 | +4.6 | 0.63 (0.36-1.12) |  |
| **High-risk^$^ population** | 93.1 (89.9-96.6) | 92.9 (89.5-96.4) | -0.2 | -0.2 |  | 0.03 |
| High-risk - CEA ≤ 2.35 | 95.5 (92.3-98.8) | 92.5 (88.6-96.7) | -3 | -3.1 | 1.18 (0.73-1.92) |  |
| High-risk - CEA > 2.35 | 87 (78.4-96.5° | 93.6 (86.9-100) | +6.6 | +7.6 | 0.44 (0.21-0.95) |  |
| **Low-risk^$$^ population** | 95.9 (93.4-98.6) | 94.8 (92-97.7) | -1.1 | -1.1 |  | 1 |
| Low-risk - CEA ≤ 2.35 | 98.8 (97.2-100) | 95.7 (92.7-98.9) | -3.1 | -3.1 | 1.34 (0.77-2.36) |  |
| Low-risk - CEA > 2.35 | 90.3 (81.8-99.8) | 91.4 (84.4-98.9) | +1.1 | +1.2 | 1.28 (0.47-3.45) |  |

*$ T4, tumor perforation, or fewer than 10 lymph nodes examined*

*$$ T1-3 and no tumor perforation and 10 or more lymph nodes examined*

**HR for treatment effect are comparing the addition of oxaliplatin to LV5FU2 regimen*

NOTE. Absolute difference at time X gives the difference between percentages observed in the two treatment arms at time X; the relative difference at time X

gives the proportion of increase or decrease in survival rate of one arm relative to the other arm at time X.

*Absolute difference reflects a comparison of survival between the FOLFOX and LV5FU2 arms.

†Relative difference reflects a ratio of the observed survival in the FOLFOX arm and the LV5FU2 arm [(X year OS rate in the FOLFOX group _ X year OS rate in

the LV5FU2 group)/(X year OS rate in the LV5FU2 group) _ 100]

**SupTable S3:** The 3-year DFS and OS according to treatment arm and a CEA level of 2.77 ng/mL

|  |  |  |  |  | **Disease-free survival** | | | | | |  | **Overall survival** | | | | | |
| --- | --- | --- | --- | --- | --- | --- | --- | --- | --- | --- | --- | --- | --- | --- | --- | --- | --- |
|  | ***N*** | **LV5FU2**  **arm**  ***N*** | **FOLFOX**  **arm**  ***N*** |  | | |  |  |  | ***P* value for the inteaction term between CEA (≤ 2.77, >2.77) and Treatment Arm (LV5FU2, FOLFOX)** |  | | |  |  |  | ***P* value for the inteaction term between CEA (≤ 2.77, >2.77) and Treatment Arm (LV5FU2, FOLFOX)** |
|  |  |  |  | **LV5FU2**  **arm**  **95% CI** | | **FOLFOX**  **arm**  **95% CI** | **Absolute change** | **Relative change** | **HR for treatment effect***  **(95%CI)** |  | **LV5FU2**  **arm**  **95% CI** | | **FOLFOX**  **arm**  **95% CI** | **Absolute change** | **Relative change** | **HR for treatment effect* (95%CI)** |  |
| **Whole population** | 899 | 448 | 451 | 84.7 (81.4-88.1) | | 87.1 (84-90) | +2.4 | +2.8 |  | 0.26 | 94.6 (92.5-96.7) | | 93.7 (91.5-96) | -0.9 | -0.9 |  | 0.11 |
| - CEA ≤ 2.77 | 724 | 360 | 364 | 87.7 (84.4-91.2) | | 88.9 (85.7-92.2) | +1.2 | +1.4 | 1.01 (0.74-1.38) |  | 96.6 (94.8-98.5) | | 94.2 (91.8-96.6) | -1.8 | -1.9 | 1.20 (0.84-1.7) |  |
| - CEA > 2.77 | 143 | 70 | 73 | 73.9 (64.2-85.1) | | 76.7 (67.5-87) | +2.8 | +3.8 | 0.72 (0.41-1.26) |  | 88.3 (81-96.3) | | 90.4 (83.9-97.4) | +2.1 | +2.4 | 0.68 (0.37-1.28) |  |
| **High-risk^$^ population** | 434 | 222 | 212 | 81.3 (76.2-86.6) | | 86.3 (81.7-91) | +5 | +6.1 |  | 0.09 | 93.1 (89.9-96.6) | | 92.9 (89.5-96.4) | -0.2 | -0.2 |  | 0.097 |
| High-risk - CEA ≤ 2.77 | 353 | 174 | 179 | 83.7 (78.4-89.4) | | 87.1 (82.3-92.1) | +3.4 | +4.1 | 0.92 (0.61-1.38) |  | 94.8 (91.5-98.2° | | 93.3 (89.6-97) | -1.5 | -1.6 | 1.10 (0.68-1.72) |  |
| High-risk - CEA > 2.77 | 69 | 39 | 30 | 71 (57.9-87.1) | | 83.3 (71-97.8) | +12.3 | +17.3 | 0.45 (0.20-1.04) |  | 86.7 (76.6-98.3) | | 90 (79.9-100) | +3.3 | +3.8 | 0.48 (0.21-1.13) |  |
| **Low-risk^$$^ population** | 458 | 223 | 235 | 87.9 (83.7-92.3) | | 88 (83.9-92.3) | +0.1 | +0.1 |  | 0.78 | 95.9 (93.4-98.6) | | 94.8 (92-97.7) | -1.1 | -1.1 |  | 0.85 |
| Low-risk - CEA ≤ 2.77 | 365 | 183 | 182 | 91.2 (87.2-95.4) | | 91.1 (87.1-95.4) | -0.1 | -0.1 | 1.10 (0.68-1.79) |  | 98.4 (96.5-100) | | 95.6 (92.6-98.6) | -2.8 | -2.8 | 1.30 (0.75-2.25) |  |
| Low risk - CEA > 2.77 | 73 | 31 | 42 | 77.4 (64-93.6) | | 71.3 (58.9-86.5) | -6.1 | -7.9 | 1.18 (0.52-2.71) |  | 90.3 (80.5-100) | | 90.5 (82-99.8) | +0.2 | +0.2 | 1.41 (0.48-4.12) |  |

*$ T4, tumor perforation, or fewer than 10 lymph nodes examined*

*$$ T1-3 and no tumor perforation and 10 or more lymph nodes examined*

**HR for treatment effect are comparing the addition of oxaliplatin to LV5FU2 regimen*

NOTE. Absolute difference at time X gives the difference between percentages observed in the two treatment arms at time X; the relative difference at time X

gives the proportion of increase or decrease in survival rate of one arm relative to the other arm at time X.

*Absolute difference reflects a comparison of survival between the FOLFOX and LV5FU2 arms.

†Relative difference reflects a ratio of the observed survival in the FOLFOX arm and the LV5FU2 arm [(X year OS rate in the FOLFOX group _ X year OS rate in

the LV5FU2 group)/(X year OS rate in the LV5FU2 group) _ 100]

**SUPPLEMENTARY FIGURES**

**Sup Figure S1:** OS according to (A) post-operative CEA 5 ng/mL and (B) post-operative CEA 2.35 ng/mL

**Supp Figure 2:** DFS (A) and OS (B) according to CEA cut-off defined with the Hothorn and Lausen method.

**Supp Fig S3:** Benefit for DFS and OS with the addition of oxaliplatin to LV5FU2 in (A and D, respectively) all patients, in low-risk patients (B and E, respectively), and high-risk patients (C and F, respectively) according to a CEA cut-off of 2.77 ng/mL.

*Ox: oxaliplatin*

**Supp Fig S4:** Benefit DFS with the addition of oxaliplatin to LV5FU2 (A) in modified MOSAIC low-risk patients, (B) in modified MOSAIC low-risk patients with CEA stratification, (C) in modified MOSAIC high-risk patients, and (D) in modified MOSAIC high-risk patients with CEA stratification.

****.

**Supp Figure S5:** Benefit OS with the addition of oxaliplatin to LV5FU2 (A) in modified MOSAIC low-risk patients, (B) in modified MOSAIC low-risk patients with CEA stratification, (C) in modified MOSAIC high-risk patients, and (D) in modified MOSAIC high-risk patients with CEA stratification.

**

**Supp Figure S6:** Benefit DFS with the addition of oxaliplatin to LV5FU2 in (A) MOSAIC low-risk patients ≤70 years, (B) in MOSAIC low-risk patients ≤70 years with CEA stratification, (C) in MOSAIC high-risk patients ≤70 years, and (D) in MOSAIC high-risk patients ≤70 years with CEA stratification.

**

**Supp Figure S7:** Benefit OS with the addition of oxaliplatin to LV5FU2 in (A) MOSAIC low-risk patients ≤70 years, (B) in MOSAIC low-risk patients ≤70 years with CEA stratification, (C) in MOSAIC high-risk patients ≤70 years, and (D) in MOSAIC high-risk patients ≤70 years with CEA stratification.

**
